# Prevalence of intimate partner violence among child marriage victims and the comparison with adult marriages: a systematic review and meta-analysis

**DOI:** 10.1016/j.eclinm.2025.103084

**Published:** 2025-02-13

**Authors:** Qing Han, Wenting Ye, Zuyi Fang, Stephanie Eagling-Peche, Yuwei Wang, Bang Zheng, Jamie Lachman

**Affiliations:** a Department of Social Policy and Intervention, University of Oxford, Oxford, UK; b Faculty of Psychology, Southwest University (SWU), Chongqing, China; c Institute of Population Research, Peking University, Beijing, China; d Department of Global Health, University of Washington, Seattle, USA; e Department of Epidemiology & Biostatistics, School of Public Health, Peking University, Beijing, China; f Department of Non-communicable Disease Epidemiology, London School of Hygiene & Tropical Medicine, London, UK; g Ageing Epidemiology Research Unit, School of Public Health, Imperial College London, London, UK; h Key Laboratory of Epidemiology of Major Diseases (Peking University), Ministry of Education, Beijing, China; i Centre for Social Science Research, University of Cape Town, Cape Town, South Africa

**Keywords:** Child marriage, Intimate partner violence, Prevalence, Meta-analysis

## Abstract

**Background:**

The global prevalence of child marriage remains high. This systematic review and meta-analysis aims to provide global estimates of the prevalence of intimate partner violence (IPV) among women who are, or have been, child brides (i.e., child marriage population), and the relative risks compared with the adult marriage population.

**Methods:**

We searched PubMed, Embase, APA PsycArticles, APA PsycInfo, Web of Science Core Collection, EBSCO, and ProQuest Dissertations & Theses Global for studies published from database inception to November 6, 2024. Eligible studies that reported IPV data either in the child marriage population or in both child marriage and adult marriage populations were included. Random effects meta-analyses were conducted to synthesise the data. The study protocol was registered on PROSPERO (CRD42023408835).

**Findings:**

A total of 16 studies on IPV experienced in the past 12 months among child brides (N = 232,928) and 23 studies on ever-experienced IPV (N = 196,929) were included. The lifetime prevalence and 12-month prevalence of any IPV in women who underwent child marriage were 35% (95% CI: 28–43) and 24% (95% CI: 16–31), respectively. Compared with women married in adulthood, women married as children had substantially higher odds of experiencing any IPV over lifetime (OR = 1.42, 95% CI: 1.22–1.65) or in the past 12 months (OR = 1.37, 95% CI: 1.18–1.57). The excess risks persisted even after child brides reached adulthood. Consistent findings were obtained after restricting to nationally representative data.

**Interpretation:**

Women who underwent child marriage are vulnerable to and disproportionally affected by IPV, emphasising the need for international efforts on targeted IPV interventions and ending child marriage globally to prevent more victims.

**Funding:**

None.

**Research in context**

**Evidence before this study**

We searched PubMed, Embase, APA PsycArticles, APA PsycInfo, Web of Science Core Collection, EBSCO, and ProQuest Dissertations & Theses Global for relevant studies published from inception to November 6, 2024 using the search terms related to child marriage and intimate partner violence. A growing body of data suggest child brides face heightened vulnerability to IPV, but no meta-analysis has yet been done to obtain estimates of IPV prevalence and excess risks in this vulnerable population. One previous systematic review on the health consequences of child marriage revealed a higher likelihood of experiencing physical IPV, but found limited evidence of sexual IPV.

**Added value of this study**

In this comprehensive systematic review and meta-analysis involving a total of 395,113 women married as children across five continents, we found a high burden of IPV in this vulnerable population (35% for lifetime prevalence and 24% for 12-month prevalence). Their odds of experiencing IPV were substantially higher than the adult marriage comparators (OR = 1.42, 95% CI: 1.22–1.65 over lifetime and 1.37, 95% CI: 1.18–1.57 in the past 12 months), especially for physical IPV. In addition, such excess risks persisted even after child brides reached adulthood, highlighting the need for targeted interventions.

**Implications of all the available evidence**

This systematic review and meta-analysis based on up-to-date global data revealed a high prevalence of IPV among women who underwent child marriage, of which the risk was significantly higher than that in the adult marriage comparators. The substantial IPV burden and the lifelong disproportional impact emphasise the need for international efforts on targeted IPV interventions in this vulnerable population, and the urgency of ending child marriage globally to prevent more victims.

## Introduction

The United Nations Children’s Fund (UNICEF) defines child marriage as: “any legal or customary union involving a boy or girl below the age of 18”.^1^ Despite the increased prioritisation of human rights entities and governments in recent years to end child and forced marriage, the global prevalence of child marriage remains high. It has been estimated that over 650 million women alive today were married as children, and according to the UNICEF’s global databases in 2022, there are still 12 million new child brides every year.^2^ To make matters worse, the COVID-19 pandemic was estimated to increase the number of child brides by an additional tenth through pathways such as economic insecurity and school closure.^3^ While boy marriages also occur, child marriages are often the result of deep-rooted gender inequalities that make girls the main victims. Globally, the child marriage rate for boys is only one-sixth of that for girls.^2^ When a girl becomes a bride, it is undoubtedly a fundamental violation of human rights and a form of violence against girls.

Unfortunately, child marriage is often just the beginning of a series of social and health threats and adverse events, of which a common type experienced by child brides is intimate partner violence (IPV).^4^^,^^5^ Empirical data suggests that child marriage could lead to a higher risk of IPV.^4^ Reasons for higher vulnerability to IPV among women who underwent child marriage could be multi-fold, including educational deprivation after marriage, lack of social support and economic resources, and power disparities in the relationship due to age gaps or economic dependence.^6^ Child brides are often exposed to various forms of violence from their husbands or partners, which can include physical violence, sexual violence and emotional violence.^7^

Over the last decade, there has been a growing body of data on IPV among child marriage populations (e.g., from the Demographic and Health Surveys, DHS), mostly at the national or sub-national level.^8^, ^9^, ^10^ However, no meta-analysis has yet been done to obtain global estimates of IPV prevalence and excess risks in this vulnerable population. Quantitative syntheses of existing evidence at the global level are urgently needed to provide timely estimates of the IPV burden in this population to inform resource allocations for targeted interventions.

Therefore, we conducted a comprehensive systematic review and meta-analysis to: (1) estimate the prevalence of major types of IPV (physical, emotional, sexual, or any) across different timeframes (last 12 months or lifetime) among the child marriage population around the globe; (2) compare IPV risks between the child marriage population and the adult marriage population; and (3) explore factors influencing the child marriage-related IPV burden, and whether child marriage victims continue to experience excess IPV burden as they reach adulthood. In this study, we defined the child marriage population as people who were married as children, including those under age 18 who have already married, and the adults who married in childhood.^2^ With these quantitative evidence syntheses, this study aims to provide support for the achievement of the fifth UN Sustainable Development Goal - achieving gender equality, empowering all girls, and addressing intimate partner violence.^11^

## Methods

### Search strategy

We conducted a systematic search of seven international databases on November 6, 2024: PubMed, Embase, APA PsycArticles, APA PsycInfo, Web of Science Core Collection, EBSCO and ProQuest Dissertations & Theses Global (updated following an initial search on August 1, 2022). We searched for studies that reported IPV data either in the child marriage population or in both child marriage and adult marriage populations. Search terms related to child marriage and intimate partner violence were used. Reference lists of relevant papers were checked for additional studies. Detailed search strategies for each database are presented in Appendix 1 (p 2–3).

The study protocol was registered on PROSPERO (CRD42023408835). The reporting of this study followed the Preferred Reporting Items for Systematic Reviews and Meta-analyses (PRISMA) reporting guideline.^12^

### Ethics

Not applicable for the systematic review and meta-analysis.

### Inclusion and exclusion criteria

Two authors (QH and BZ) independently screened studies for inclusion using EndNote X9, with discrepancies settled after consensus discussions with another author (WY). Articles were included if they reported cross-sectional or longitudinal data on the prevalence of IPV among the child marriage population (regardless of whether they had reached adulthood before the data collection), and/or the comparison with the adult marriage population. Qualitative studies or interventional studies (unless baseline data were available) were excluded. According to the World Health Organization (WHO)’s definition,^13^ IPV includes physical, sexual, and emotional abuse, as well as controlling behaviours by an intimate partner. In our study, eligible articles should have assessed at least one type of IPV perpetrated by intimate partners, such as husbands and cohabiting partners, over the past 12 months or lifetime before data collection. No restrictions on participants’ age, geographic coverage, or study settings (e.g., population-based household survey, hospital-based study, or online survey) were set when assessing the eligibility of the studies, unless the sample was selected in a biased manner (e.g., a survey among victims of domestic violence). Studies without representative samples were included in the main analysis but excluded later in a sensitivity analysis. Studies focusing on general family violence instead of IPV, not reporting measurement methods for IPV, using IPV definitions that were incomparable with the majority of studies, or not specifying types of IPV measured were excluded. We did not include economic IPV due to insufficient data. Studies in the same country but with different data sources were included. For studies with overlapping data sources (e.g., two studies using data from the same survey as part of their analyses), only the study with the largest sample size was included unless there was a concern over its study quality; however, we kept all studies that used the same data source but reported different types of IPV, as our analyses were stratified by IPV type.

For the estimation of IPV prevalence among the child marriage population, eligible articles were required to provide prevalence estimates for individuals who have experienced child marriage and the sample sizes. Studies should have collected the age at which participants entered into marriage or cohabitation and provided clear criteria for the identification of child marriage cases.

For the estimation of relative risks of experiencing IPV between people married as children vs. as adults, eligible articles were required to report the odds ratio (OR), or sufficient data for the calculation of OR (e.g., IPV prevalence and sample size for both child marriage and adult marriage populations under investigation).

### Data extraction and risk of bias assessment

Two authors (QH and WY) independently extracted data and assessed the risk of bias, with discrepancies resolved after consensus discussions with another author (BZ). A standardised data extraction form was created and included the following information: study design, study area, data source, participant characteristics, child marriage definition (or age range), sample size of child marriage population, types of IPV, measurement instrument and timeframe, and the prevalence or proportion of experiencing IPV in this population. For the estimation of relative risks, additional information was extracted: sample size of the adult marriage population, prevalence or proportion of experiencing IPV in this population, and adjusted (or if not available, unadjusted) odds ratio and its standard error (SE) or confidence interval (CI).

The quality of included papers was evaluated using the Joanna Briggs Institute (JBI) Critical Appraisal Checklist for Studies Reporting Prevalence Data.^14^ We assigned an overall quality rating of high, moderate or low to each study after qualitative evaluation based on the nine items in the checklist.

### Statistics

To illustrate the latest IPV burden among child marriage populations across countries, we plotted the 12-month IPV prevalence in a world map based on data from a subset of included papers which had nationally representative samples. When there were multiple nationally representative studies for a single country, we only presented the latest one in this descriptive plot.

Random-effects meta-analyses were conducted to synthesise the evidence given the heterogeneity in the data. (1) To estimate the pooled IPV prevalence and its 95% CI among women who experienced child marriage, prevalence estimates from individual studies were combined using a random-effect meta-analysis stratified by timeframe of IPV measurement (i.e., 12-month or lifetime) and IPV subtypes: physical violence, emotional violence, sexual violence, or any combination of these subtypes (referred to as “any IPV” hereafter). (2) For studies that reported data for both child marriage and adult marriage populations, another random-effect meta-analysis was conducted to estimate the relative risks of experiencing IPV between these two groups by combining odds ratios from individual studies, also stratified by IPV types and timeframe. Heterogeneity across studies was assessed using the Q test and I^2^ statistic. For each meta-analysis of relative risk estimates with at least 10 studies,^15^ a funnel plot was created to visually assess the publication bias, followed by an Egger’s test of asymmetry. Similarly, the Doi plot and LFK test were used to assess the publication bias for meta-analyses of prevalence estimates.

To examine the robustness of the main findings, several sensitivity analyses were performed by: (1) restricting to studies with nationally representative sampling; (2) restricting to studies rated as high quality; (3) restricting the relative-risk meta-analysis to studies reporting adjusted estimates (mainly controlling for sociodemographic covariates); (4) restricting the relative-risk meta-analysis to studies specifically adjusting for age at the survey or studies only including participants of similar age (e.g., 20–24 years old) to disentangle the effects of current age and age at marriage (as IPV risk also differs by current age); and (5) repeating the prevalence meta-analyses using prevalence estimates after logit transformation or Freeman–Tukey double-arcsine transformation.

Additionally, we conducted univariable meta-regressions to explore potential influencing factors, including country-level prevalence rate or absolute number of child marriages, country income level (low-income vs. high- or middle-income countries), and country-level female Gross National Income (GNI) per capita. We also conducted a subgroup analysis restricted to studies that measured IPV after the child marriage victims reached adulthood. We planned to explore the influence of legal marriage age for girls but did not conduct the analysis due to low variation in this variable.

The analyses were conducted using the R package *meta*.^16^ All statistical tests were two-sided with the significance threshold of *P* < 0.05.

### Role of funding source

There was no funding source for this study. The corresponding author had full access to all the data in the study and had final responsibility for the decision to submit to publication.

## Results

**Fig. 1:**
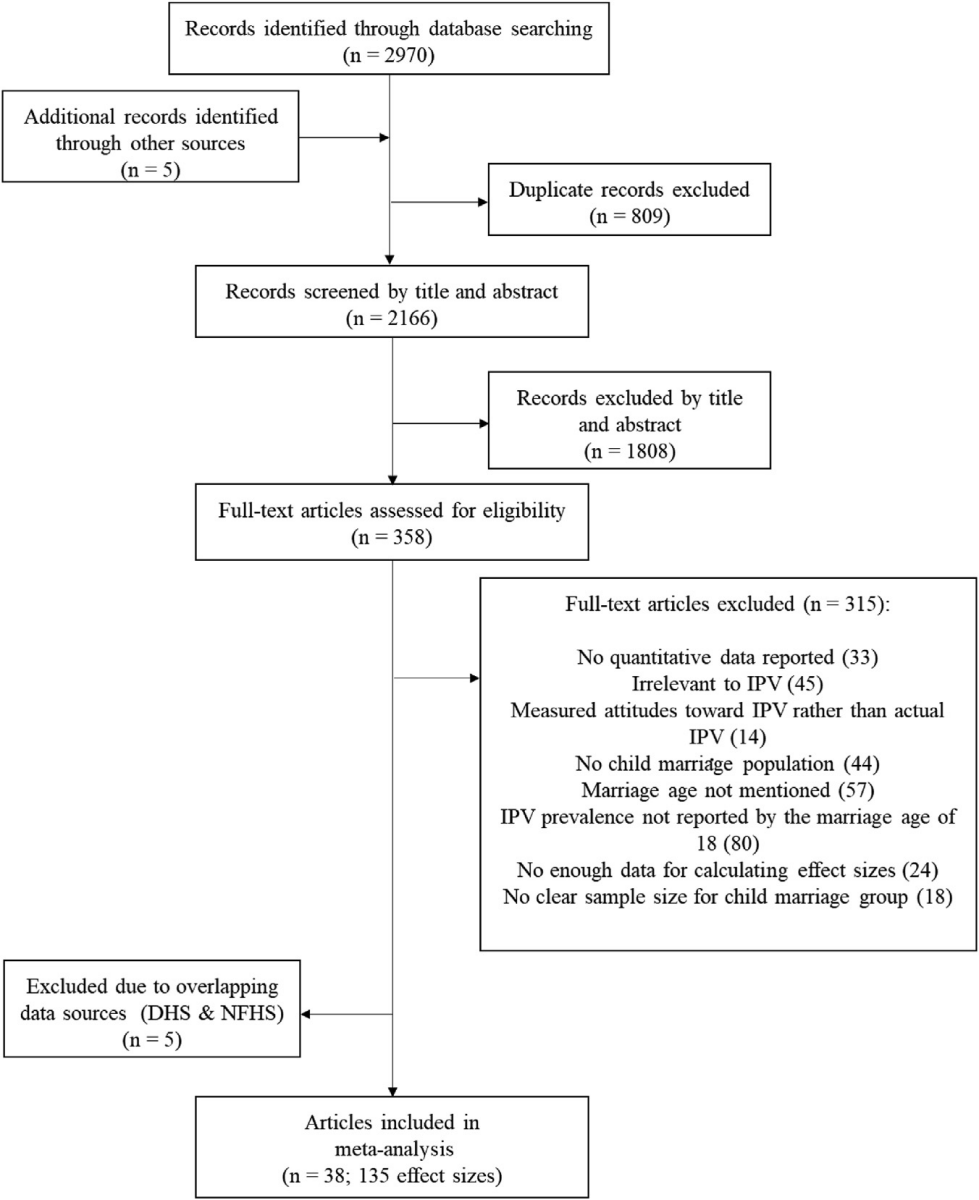
**PRISMA flowchart.** Note: IPV = intimate partner violence; DHS = Demographic and Health Survey; NFHS = National Family Health Survey. The number of effect sizes refers to the total count of prevalence and odds ratio estimates.

The literature search in seven databases yielded 2970 records. After the screening of title/abstract and full text and the detection of overlapping data, 38 eligible studies involving a total of 395,113 women married as children were included in the meta-analyses (Fig. 1). No eligible studies reported data on IPV among males married as children.

**Fig. 2:**
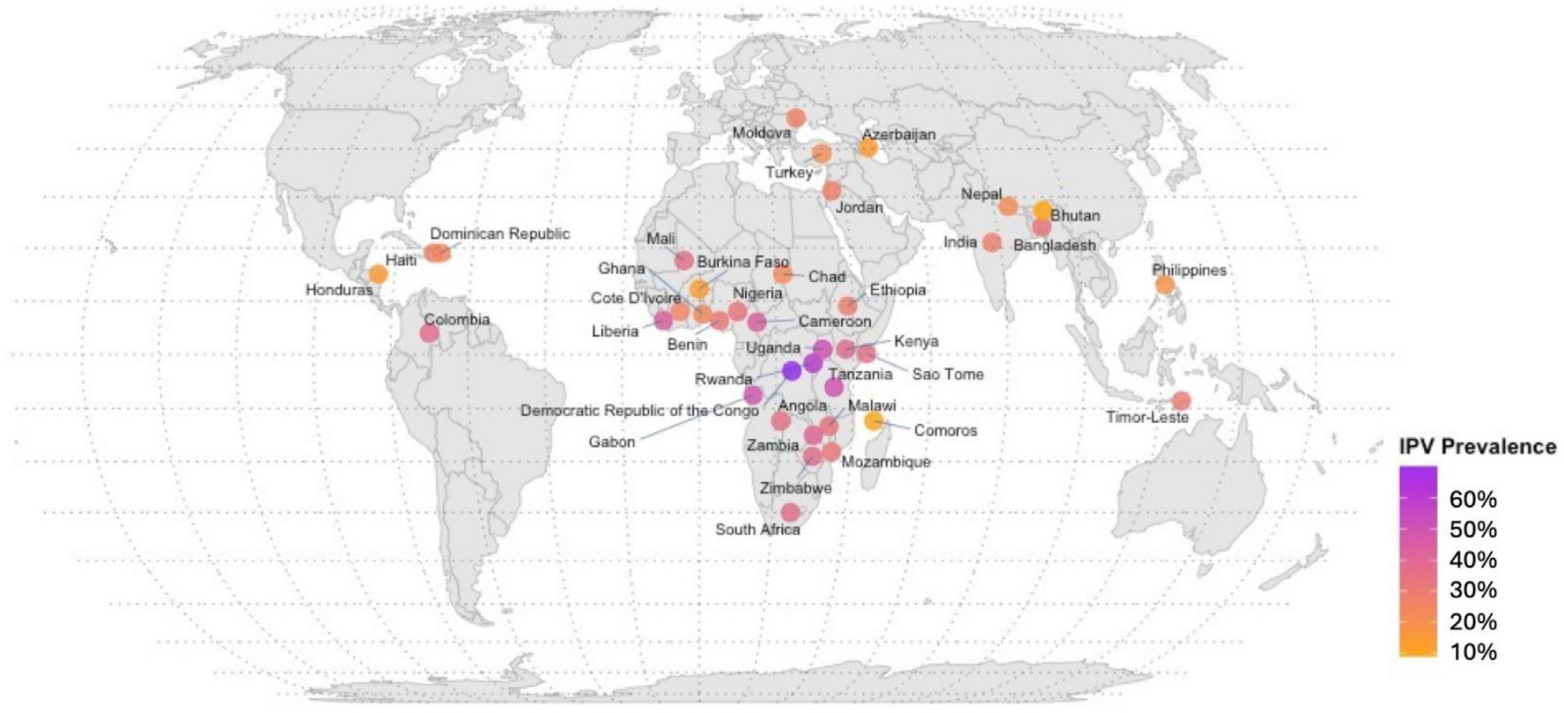
**Global distribution of any IPV in the past 12 months among child marriage populations based on nationally representative data from studies included in the meta-analysis.** Note: If there were more than one representative sample from the same country, the most recent data were used in this plot.

Table 1 describes the study characteristics and data sources of the 38 included studies. Of these 38 studies, 16 measured IPV in the past 12 months and 23 measured ever-experienced IPV (of which one study measured both). All the included studies measured at least one of the following types of IPV: physical (number of studies: k = 27, number of child brides: N = 141,560), sexual (k = 20, N = 141,127), emotional (k = 15, N = 79,635), or any (k = 20, N = 308,933, a composite measure defined as physical/sexual/emotional violence or physical/sexual violence by most studies). Most studies used an adapted or modified version of the Revised Conflict Tactics Scale^50^ (k = 22, N = 378,727) or WHO’s Multi-Country Study on Women’s Health and Domestic Violence against Women questionnaire^51^ (k = 5, N = 4855) for IPV measurement which were generally similar in item dimensions and the response form; the other 11 studies (N = 11,531) used survey questionnaire or structured interview with comparable content and format (Table 1). 23 studies (N = 381,812) used nationally representative data, and 18 (N = 374,667) were based on the Demographic and Health Surveys Program. Based on the JBI checklist,^14^ 21 (N = 381,174), 10 (N = 12,649) and 7 (N = 1290) studies were rated as high, moderate and low quality, respectively, with the common sources of bias being sampling strategy and IPV measurement instrument. Detailed critical appraisal results are displayed in Appendix 1 p 4–6. The studies covered 65 countries across the globe, with 15 studies in Africa (N = 78,254), 17 in Asia (N = 173,071), three in Europe (N = 499), one in North America (N = 425) and two based on data from multiple countries in South America/Asia/Europe/Africa (N = 142,864). The data collection time ranged from 1997 to 2021, but the data collected within the last decade accounted for 55.3% of the total number of included studies. Fig. 2 illustrates the prevalence of any IPV over the past 12 months among child marriage populations in different countries, based on nationally representative samples from the papers included in the meta-analysis.

**Table 1:** Characteristics of studies included in the meta-analysis.

Author (year)	Data source	Area	Representative	IPV timeframe	IPV type	Child marriage age	Sample size (child marriage)	Sample size (adult marriage)	IPV measurement
Yount et al. (2016)^17^	A stratified, multistage sampling survey	Bangladesh	Yes	Last 12 months	Physical	<18	2312	1043	Adapted from the Revised Conflict Tactics Scale
Jabbi et al. (2020)^18^	Demographic and Health Survey (DHS) 2013	Gambia	Yes	Last 12 months	Physical, sexual, emotional	<18	1653	1385	Shortened and modified version of the Conflict Tactics Scale
Gebrezgi et al. (2017)^19^	Facility-based cross-sectional study	Northern Ethiopia	No	Last 12 months	Physical violence during pregnancy	<18	160	262	Pretested semi-structured locally adapted questionnaire based on the World Health Organization (WHO) Multi-Country Study on Women’s Health and Domestic Violence
Yüksel-Kaptanoğlu et al. (2012)^20^	National Research on Domestic Violence against Women in Turkey	Turkey	Yes	Last 12 months	Any (physical/sexual)	<18	205	12,590	WHO’s Multi-Country Study on Women’s Health and Domestic Violence
Ahinkorah et al. (2021)^21^	DHS 2015–2019	Sub-Saharan Africa-16 countries	Yes	Last 12 months	Physical, emotional, sexual, any	<18	7092	6928	Modified Conflict Tactics Scale
Kidman (2017)^22^	DHS 2005–2013	33 countries (India excluded due to overlapping data)	Yes	Last 12 months	Physical, sexual, any	<18	14,205	15,179	Modified Conflict Tactics Scale
Kimuna et al. (2012)^23^	National Family Health Survey-3 (NFHS-3), part of the DHS program	India	Yes	Last 12 months	Physical, sexual	<18	34,946	34,538	Modified version of the Revised Conflict Tactics Scale
Phuntsho et al. (2022)^24^	2012 National Health Survey	Bhutan	Yes	Last 12 months	Physical, emotional, sexual, any	<18	3990	8220	Adapting the WHO’s Multi-Country Study on Women’s Health and Domestic Violence against Women questionnaire
Speizer & Pearson (2011)^25^	National Family Health Survey-3 (NFHS-3), part of the DHS program	India	Yes	Last 12 months and lifetime	Any (physical/sexual)	<18	34,744	25,097	Modified version of the Revised Conflict Tactics Scale
Falb et al. (2015)^26^	Baseline data of a randomised controlled trial on changing intimate partner violence	Côte d’Ivoire	No	Last 12 months	Physical, sexual, emotional, any (physical/sexual)	<18	202	480	WHO Multi-Country Study on Women’s Health and Domestic Violence
Islam (2021)^27^	A cross-sectional survey in Kutupalong Refugee makeshift	Bangladesh	No	Last 12 months	Physical, sexual	<18	298	188	Modified Conflict Tactics Scale
Kidman & Heymann (2018)^7^	DHS 1997–2015	47 countries	Yes	Last 12 months	Any (physical, sexual or emotional)	<18	128,659	209,918	Modified Conflict Tactics Scale
Kiragu et al. (2022)^28^	DHS 2014	Kenya	Yes	Last 12 months	Physical violence during pregnancy	<18	1128	2065	Modified Conflict Tactics Scale
Rahman et al. (2014)^10^	DHS 2007	Bangladesh	Yes	Last 12 months	Physical, sexual, any (physical/sexual)	<18	1630	544	Shortened and modified Conflict Tactics Scale
El-Gazzar et al. (2020)^29^	Household survey	Rural Upper Egypt	No	Last 12 months	Physical, sexual	<18	654	75	Multiple-item questionnaire (exposed to physical violence if responding “Yes” to any of the 6 items, e.g., being kicked, dragged, or beaten up; exposed to sexual violence if responding “Yes” to any of the 3 items, e.g., practicing sex against will)
Silverman et al. (2023)^30^	Baseline survey as part of a cluster-randomised trial	Niger	No	Last 12 months	Any (physical/sexual)	≤19	1050	–	Modified version of the Revised Conflict Tactics Scale
Santhya et al. (2010)^31^	A large-scale survey of young people	India	No	Lifetime	Physical, sexual	<18	5277	3037	Structured interview (e.g., whether her husband had ever slapped, punched, kicked, dragged, choked, pushed, shaken or beaten her; twisted her arm; whether her husband had ever forced her to engage in sex)
Nasrullah et al. (2014)^32^	DHS 2012–2013	Pakistan	Yes	Lifetime	Physical, emotional, any (physical/emotional)	<18	297	292	Shortened and modified version of the Conflict Tactics Scale
Qamar et al. (2022)^33^	DHS 2018	Afghanistan	Yes	Lifetime	Physical, emotional, sexual, any	<18	7483	10,740	Modified version of the Conflict Tactic Scale
Verma & Nair (2022)^34^	DHS 2014	Egypt	Yes	Lifetime	Physical, emotional, sexual, any	<18	1617	4669	Adapted using the Revised Conflict Tactics Scale
Abera et al. (2020)^8^	A community-based cross-sectional survey	Western Amhara region, Ethiopia	No	Lifetime	Physical, sexual, emotional	<18	444	834	Survey questionnaires (ever verbally abused, beaten, or forced for sex by first husband)
Olamijuwon et al. (2017)^35^	DHS 2010–2015	Sub-Saharan Africa-18 countries	Yes	Lifetime	Sexual	<18	4308	–	Modified version of the Conflict Tactic Scale
Clark et al. (2017)^36^	Jordan Population and Family Health Survey (JPFHS), part of the DHS program	Jordan	Yes	Lifetime	Any (physical/sexual)	<18	1229	4954	Revised Conflict Tactics Scale
Durğut & Kısa (2018)^37^	A hospital-based survey	Turkey	No	Lifetime	Physical	<18	246	–	Survey questionnaires (presence of physical violence)
Gubi et al. (2020)^38^	DHS 2016	Uganda	Yes	Lifetime	Physical, emotional, sexual	<18	3299	3580	Shortened and modified version of the Conflict Tactics Scale
Mondal & Paul (2021a)^39^	National Family Health Survey-4 (NFHS-4), part of the DHS program	India	Yes	Lifetime	Physical, sexual, emotional	<18	26,756	37,630	Modified version of the Revised Conflict Tactics Scale
Mondal & Paul (2021b)^9^	National Family Health Survey-4 (NFHS-4), part of the DHS program	India	Yes	Lifetime	Any (physical, sexual or emotional)	<18	26,870	37,681	Modified version of the Revised Conflict Tactics Scale
Oshiro et al. (2011)^40^	A community-based, cross-sectional study in Kathmandu Metropolitan City	Nepal	No	Lifetime	Physical	<18	298	607	WHO’s Multi-Country Study on Women’s Health and Domestic Violence
Tenkorang (2019)^6^	Nationally representative cross-sectional data	Ghana	Yes	Lifetime	Physical, emotional, sexual	<18	213	2076	Structured interview (experienced physical violence if they answered in the affirmative to at least one of the 5 questions, e.g., pushed, shook or threw something at them; sexual violence with 3 questions, e.g., physically forced to have sex with them even when they did not want to; emotional violence with 3 questions, e.g., said or did something to humiliate them in front of others)
Valan & Srinivasan (2021)^41^	A structured interview	India	No	Lifetime	Physical, emotional	<18	252	–	Structured interview (whether faced verbal abuse, psychological abuse, and physical abuse)
Erulkar (2013)^42^	A population-based survey	Seven Ethiopian regions	No	Lifetime	Physical, sexual	<18	771	432	Survey questionnaires (whether the consummation of her marriage had been forced or consensual; whether her husband had ever been physically violent with her, i.e., had pushed, slapped, punched, beaten or kicked her)
Begum et al. (2015)^43^	A community-based cross-sectional household survey	India	No	Lifetime	Any (physical, sexual or emotional)	<18	400	737	Shortened and modified Conflict Tactics Scale
Huber-Krum et al. (2023)^44^	2017 Honduras Violence Against Children and Youth Survey (VACS)	Honduras	Yes	Lifetime	Any (physical/sexual)	<18	425	–	Survey questionnaires (reporting at least one of the 4 items of sexual IPV or 5 items of physical IPV)
Mehari et al. (2023)^45^	A cross-sectional survey	Eritrea	No	Lifetime	Any	<18	84	116	Survey questionnaires (presence of spousal violence)
Verma & Choudhury (2023)^46^	National Family Health Survey-5 (NFHS-5), part of the DHS program	India	Yes	Lifetime	Physical, emotional, sexual, any (physical, sexual, emotional IPV or injuries)	<18	23,172	37,308	Modified version of the Revised Conflict Tactics Scale
Soylu & Ayaz (2013)^47^	A hospital-based study	Turkey	No	Lifetime	Physical, emotional	≤18	48	–	Social investigation (physical abuse by their spouse, e.g., slapping, punching, kicking, hitting with an object, dragging, choking; emotional abuse, e.g., insults, humiliation, or being demeaned in front of others)
Patel et al. (2024)^48^	Understanding the Lives of Adolescents and Young Adults Survey	India	No	Lifetime	Physical, emotional, sexual	≤19	3117	–	Survey questionnaires (emotional IPV: ever humiliated in front of others or threatened to hurt or harm something close to her; sexual IPV: ever forced to have sex; physical IPV: 7 items, e.g., ever slap you)
Seifu et al. (2024)^49^	DHS 2012–2021	Sub-Saharan Africa-30 countries	Yes	Lifetime	Any (physical, sexual)	≤19	55,579	31,144	Modified version of the Revised Conflict Tactics Scale

IPV = intimate partner violence; DHS = Demographic and Health Survey; WHO = World Health Organization.

### IPV prevalence among the child marriage population

**Fig. 3:**
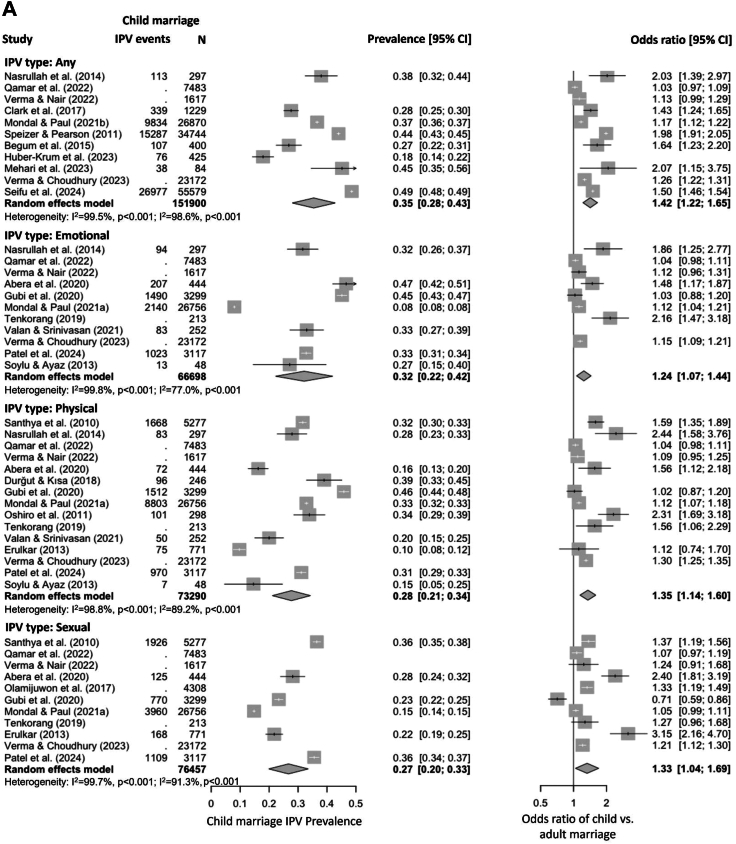

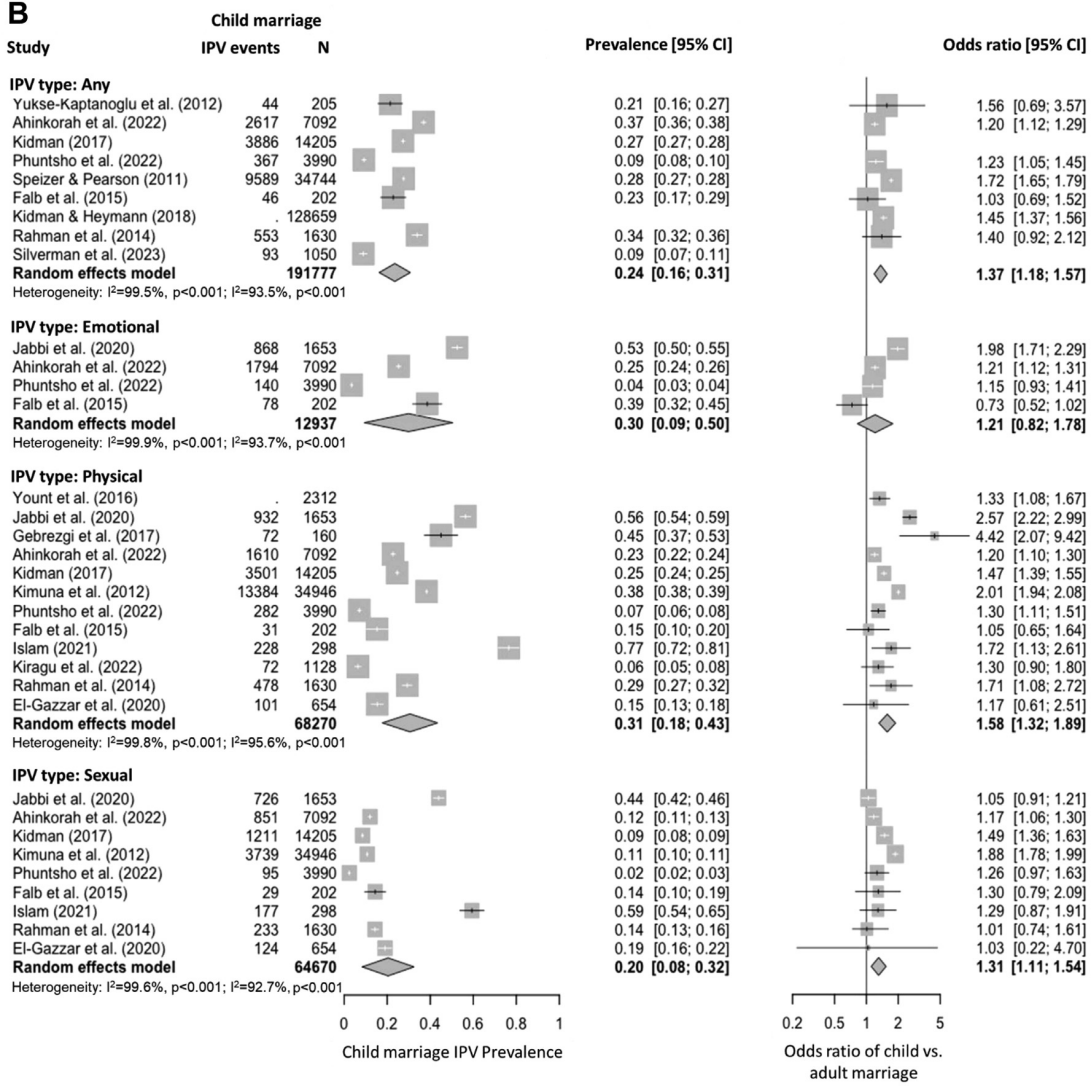
**Forest plots of the prevalence of lifetime IPV (A) and 12-month IPV (B) among the child marriage population and relative risks compared with adult marriages.** Note: Estimates from individual studies are represented by filled squares, with the size indicative of the weight in the meta-analysis. Summary estimates are represented by filled diamonds. The error bars and diamond width represent the 95% CIs of the estimates. Heterogeneity statistics are displayed for the meta-analyses of prevalence (the first set) and odds ratios (the second set) separately.

Results of the meta-analysis on the prevalence of different types of IPV over lifetime among the child marriage population (N = 196,929) are presented in Fig. 3A. The lifetime prevalence of any IPV in women who underwent child marriage was 35% (95% CI: 28–43). For the specific types of IPV, emotional IPV had a prevalence estimate of 32% (95% CI: 22–42), followed by physical IPV (28%, 95% CI: 21–34) and sexual IPV (27%, 95% CI: 20–33). In this meta-analysis of lifetime prevalence, 94.4% (185,992 out of 196,929) of participants were from nationally representative samples. Sensitivity analyses that only included nationally representative samples yielded lifetime prevalence estimates of 36% (95% CI: 27–44), 28% (95% CI: 7–50), 36% (95% CI: 25–46) and 19% (95% CI: 11–27) for any, emotional, physical and sexual IPV, respectively (Appendix 1 p 7).

The pooled prevalence of IPV over the past 12 months among the child marriage population (N = 232,928) is shown in Fig. 3B. The pooled 12-month prevalence of any IPV was lower than the lifetime prevalence, with an estimate of 24% (95% CI: 16–31). The analyses for specific types of IPV yielded even higher estimates due to different data sources being pooled, with the estimates for emotional, physical and sexual IPV being 30% (95% CI: 9–50), 31% (95% CI: 18–43) and 20% (95% CI: 8–32), respectively. In this meta-analysis of 12-month prevalence, 99.0% (230,564 out of 232,928) of participants were from nationally representative samples. Sensitivity analyses restricted to those nationally representative samples yielded 12-month prevalence estimates of 26% (95% CI: 18–34), 27% (95% CI: 0–55), 26% (95% CI: 13–39) and 15% (95% CI: 4–27) for any, emotional, physical and sexual IPV, respectively (Appendix 1 p 8).

### Comparison of IPV risks between child marriage vs. adult marriage populations

The following meta-analyses were conducted based on odds ratios for IPV between child marriage and adult marriage groups from individual studies. Compared with women married as adults, women married as children had a 42% higher odds of experiencing any IPV over lifetime (OR = 1.42, 95% CI: 1.22–1.65), and a 37% higher odds of experiencing any IPV in the past 12 months (OR = 1.37, 95% CI: 1.18–1.57). The child marriage population also faced higher risks in specific types of IPV (Fig. 3). Compared with the adult marriage population, women who underwent child marriage had a substantially higher odds of experiencing physical IPV over lifetime (OR = 1.35, 95% CI: 1.14–1.60) and in the past 12 months (OR = 1.58, 95% CI: 1.32–1.89); similar patterns were also observed for sexual IPV (OR = 1.33, 95% CI: 1.04–1.69 over lifetime and 1.31, 95% CI: 1.11–1.54 in the past 12 months). The pooled OR for emotional IPV over lifetime in child marriage vs. adult marriage populations was 1.24 (95% CI: 1.07–1.44), but the pooled OR for 12-month emotional IPV did not reach statistical significance (OR = 1.21, 95% CI: 0.82–1.78). There was substantial heterogeneity across studies in these meta-analyses, with I^2^ values ranging from 77.0% to 99.9% (*P* < 0.001). No clear signs of publication bias were observed (Appendix 1 p 9–10). Further sensitivity analyses yielded results consistent with those of the main analyses (Appendix 1 p 7–8, 11–14).

### Child brides still exhibit higher vulnerability to IPV after reaching adulthood compared with adult marriage comparators

To explore whether the excess IPV risk persisted as the child brides reached adulthood, a subgroup analysis comparing 12-month IPV risks between child marriage and adult marriage populations was conducted using a subset of studies with data collected after the participants reached 18 years old. Results showed that the child marriage population still had a significantly higher odds of any IPV in the past 12 months during adulthood compared with the adult marriage population (k = 5, OR = 1.39, 95% CI: 1.17–1.64). Their odds of physical IPV and sexual IPV also remained significantly higher compared with the adult marriage population (k = 4, OR = 1.33, 95% CI: 1.13–1.57 and k = 4, OR = 1.27, 95% CI: 1.07–1.52). Similar to the main analyses, there is limited evidence on emotional IPV (k = 2, OR = 0.97, 95% CI: 0.59–1.57).

### IPV burden in countries with the highest rates or numbers of child marriage

The top ten countries globally with the highest child marriage rates have an average child marriage rate exceeding 55%.^52^ We therefore explored whether the child brides in those countries faced a higher IPV burden. Results of meta-regression by child marriage rates showed that the 12-month IPV prevalence among child brides was not significantly different between countries in the top ten list (Niger, Central African Republic, Chad, Mali, Mozambique, Burkina Faso, South Sudan, Bangladesh, Guinea, Eritrea) or the top region (Sub-Saharan Africa) and other countries (*P* > 0.05; Table 2). Similarly, results of meta-regression by child marriage numbers^53^ did not indicate any significant differences in the 12-month IPV prevalence between the top countries and other countries (*P* > 0.05; Table 2). The relative risks of IPV between child marriage and adult marriage populations did not vary significantly by child marriage rates or child marriage numbers (*P* > 0.05; Table 2).

**Table 2:** Meta-regressions on potential influencing factors on the IPV burden in the child marriage population.

Model	Factor	IPV type	No. of studies	β	SE	*P*
Past 12-month IPV prevalence in the child marriage population	Ranking of country-level child marriage rates	Any	7	0.06	0.09	0.473
		Emotional	4	−0.06	0.29	0.833
		Physical	10	0.17	0.16	0.292
		Sexual	8	0.10	0.15	0.480
	Ranking of country-level child marriage numbers	Any	7	0.09	0.08	0.272
		Emotional	4	−0.06	0.29	0.833
		Physical	10	0.22	0.13	0.093
		Sexual	8	0.04	0.15	0.783
	Low-income country vs. else	Any	6	0.01	0.10	0.904
		Emotional	–	–	–	–
		Physical	9	0.35	0.11	0.001
		Sexual	7	0.27	0.12	0.020
	Female gross national income per capita, PPP ($1000)	Any	7	0.00	0.01	0.588
		Emotional	4	−0.09	0.03	0.002
		Physical	10	−0.11	0.04	0.013
		Sexual	8	−0.07	0.04	0.135
Relative risk of past 12-month IPV between child vs. adult marriage populations	Ranking of country-level child marriage rates	Any	6	−0.09	0.20	0.634
		Emotional	4	0.01	0.56	0.993
		Physical	11	−0.18	0.22	0.412
		Sexual	8	−0.16	0.20	0.418
	Ranking of country-level child marriage numbers	Any	6	0.18	0.19	0.327
		Emotional	4	0.01	0.56	0.993
		Physical	11	0.15	0.23	0.508
		Sexual	8	0.15	0.19	0.424
	Low-income country vs. else	Any	5	0.02	0.33	0.954
		Emotional	–	–	–	–
		Physical	10	0.35	0.22	0.104
		Sexual	7	−0.34	0.18	0.051
	Female gross national income per capita, PPP ($1000)	Any	6	0.00	0.03	0.921
		Emotional	4	−0.09	0.11	0.433
		Physical	11	−0.14	0.06	0.034
		Sexual	8	0.00	0.07	0.968

Note: “–” refers to no analysis being conducted due to low number of studies in certain categories.

### Association between national economic level or female income level and IPV among child marriage population

Results of meta-regression showed that the prevalence of 12-month physical and sexual IPV among women who experienced child marriage was significantly higher in low-income countries than in high/middle-income countries (*P* = 0.001 and 0.020, respectively; Table 2). The relative risks of IPV between child and adult marriages did not vary significantly by country income level (*P* > 0.05; Table 2). Consistently, the prevalence of 12-month physical and emotional IPV among women who experienced child marriage was significantly lower in countries with higher national female income levels (*P* = 0.013 and 0.002, respectively; Table 2). We also found that higher national female income was associated with less excess risk of physical IPV in the child marriage population vs. adult marriages (*P* = 0.034; Table 2).

## Discussion

This systematic review and meta-analysis aggregated the latest global data on the IPV burden among the child marriage population. The pooled prevalence of any IPV in women who underwent child marriage was 35% (95% CI: 28–43) over lifetime, and 24% (95% CI: 16–31) in the past 12 months. Similar estimates were obtained when restricting to nationally representative samples (36% and 26%, respectively). Compared with adult marriages, women married as children had substantially higher odds of experiencing any IPV over lifetime (OR = 1.42, 95% CI: 1.22–1.65) and in the past 12 months (OR = 1.37, 95% CI: 1.18–1.57). Importantly, subgroup analysis revealed that the excess risks persisted even after child brides reached adulthood.

One of the reasons for the heightened vulnerability of IPV among women who underwent child marriage could be educational deprivation.^4^ Child brides often struggle to continue their education within the confines of marriage. A previous study in Bangladesh showed that getting married one year earlier was associated with 0.22 reduced years of schooling.^54^ Other studies also indicated that early marriage results in lower literacy rates and contributes to girls dropping out of school,^6^^,^^55^ which inevitably reduces the potential for a career of their own and hinders their ability to achieve economic autonomy in adulthood.^6^^,^^56^^,^^57^ Child marriage also removes girls from social networks and support structures provided by schools, preventing them from receiving societal support in cases of IPV; in some regions child brides were also cut off from their families after getting married. Furthermore, the internalisation of traditional gender norms makes women believe that men have a “right” to control their behaviour, and that they must conform to these norms, even in the face of violence.^58^ In such social environments, girls who marry early are more likely to rationalise their husbands’ controlling and violent behaviour. In addition, child brides often marry much older men, further exacerbating power imbalances that foster the abuse of girls.^59^ Previous literature also suggested that husbands of child brides were likely to be less educated and hold traditional masculine ideologies compared with husbands in adult marriage.^22^^,^^60^ The afore-mentioned factors are not isolated but rather intertwined in a complex and intractable cycle that is challenging to address. It is also evident that these factors do not disappear as child brides age, indicating that the adverse impacts could be lifelong, which was supported by our findings. Therefore, interventions addressing IPV should pay extra attention to the victims of child marriage, even after they have reached adulthood.

A previous systematic review on the health consequences of child marriage revealed a higher likelihood of experiencing physical violence from an intimate partner, but found limited evidence of sexual violence.^4^ In this meta-analysis, we found that child marriage also increased the likelihood of experiencing sexual IPV. Child brides are often unable to negotiate safe sex with their husbands, making them more vulnerable to sexually transmitted diseases, including HIV, and increasing their risk of early pregnancy.^61^ Additionally, due to their immature physical development and lack of proper medical care during pregnancy and childbirth, adolescent mothers often face a higher risk of pregnancy complications.^62^ The impaired reproductive health can further increase the likelihood of IPV.^63^

This study adds another layer of evidence on the adverse consequences of child marriage. At the family level, the educational deprivation of mothers who experienced child marriage and their greater acceptance of IPV collectively contribute to the intergenerational transmission of child marriage.^64^^,^^65^ At the community level, shared values and beliefs tied to early marriage further normalise IPV within interpersonal relationships and social networks.^66^ A daughter’s early marriage is often perceived as a “solution” to economic crises in low-income countries.^67^ However, as shown in our study, child brides in these low-income countries would experience a higher risk of IPV than other countries, possibly due to the economic dependence on their husband and the enlarged power disparities. Recognising the wide spectrum of adverse effects of child marriage forms the basis of altering parents’ understanding of this issue and breaking the chain of intergenerational child marriage transmission globally.

Despite the challenging context, it is encouraging that several parenting programs and child welfare intervention measures have already achieved success in breaking the vicious cycle of intergenerational transmission of child marriage and IPV.^68^^,^^69^ Additional resources should be allocated to such interventions globally to address the current and future challenges posed by IPV in child marriage population.

This study has several limitations. The number of studies in certain subtypes of IPV and geological regions remains constrained. In the comparative analysis with adult marriages, several studies failed to control for sociodemographic factors related to child marriage, making it difficult to estimate the independent effect of child marriage on IPV risks. Nevertheless, although this endogeneity issue precludes the possibility of ascertaining a causal relationship, our estimates can still reflect the current global status of an excess IPV burden among child marriage victims. It was worth noting that child brides may be underrepresented in some studies due to the difficulty in accessing this vulnerable population. Moreover, most of the included studies combined child brides with different age at marriage into one group (married before 18 years old) and did not assess the impact of very early marriage (e.g., married before 15 years old). A paper by Coll et al.^70^ found that the IPV burden was even heavier among child brides with younger age at marriage, suggesting the heterogeneity in the vulnerability among this population. Although most studies had specified the IPV perpetrator to be husband/spouse, in some circumstances the participants might have reported IPV by non-husband partner (especially for divorced women). Furthermore, while the number of child marriages among boys is notably lower than among girls,^2^ we found a surprisingly limited body of research on the IPV burden among child grooms. Additionally, child brides are at risk of sexual and maternal health threats^62^ which could potentially exacerbate their IPV risk.^63^ While some included studies have investigated IPV during pregnancy among child brides, the sample sizes were insufficient to perform subgroup analyses.

In conclusion, this systematic review and meta-analysis based on up-to-date global data revealed a high prevalence of multiple types of IPV among women who underwent child marriage, of which the risk was significantly higher than that in the adult marriage comparators. The substantial IPV burden and the lifelong disproportional impact emphasise the need for international efforts on targeted IPV interventions in this vulnerable population, and the urgency of ending child marriage globally to prevent more victims.

## Contributors

QH and BZ designed the study. QH, WY and BZ had access to and verified the underlying data, and contributed to the acquisition, analysis, or interpretation of data. QH led the statistical analysis and drafted the first version of the manuscript. JL and BZ contributed to the supervision. All authors critically revised the draft. All authors read and approved the final version of the manuscript.

## Data sharing statement

Relevant data have been presented in the manuscript and the Appendix.

## Editor note

The Lancet Group takes a neutral position with respect to territorial claims in published maps and institutional affiliations.

## Declaration of interests

All authors declare no competing interests.
