# Supplementary material for: Prevalence of intimate partner violence among child marriage victims and the comparison with adult marriages: a systematic review and meta-analysis
Source: eClinicalMedicine. 2025 Feb 13;81:103084. doi: 10.1016/j.eclinm.2025.103084 (PMC11874713; doi:10.1016/j.eclinm.2025.103084)
Supplement: Supplementary Methods, Figs. S1–S4, Tables S1–S5 [file mmc1.docx]

**Supplementary materials**

Supplementary Methods. Search strategies.

Supplementary Table 1. Risk of bias assessment of included studies.

Supplementary Fig. 1. Sensitivity analyses of lifetime IPV after restricting to nationally representative samples.

Supplementary Fig. 2. Sensitivity analyses of 12-month IPV after restricting to nationally representative samples.

Supplementary Fig. 3. DOI plots of the prevalence meta-analyses with at least 10 studies.

Supplementary Fig. 4. Funnel plots of the relative-risk meta-analyses with at least 10 studies.

Supplementary Table 2. Sensitivity analyses after restricting to studies rated as high quality.

Supplementary Table 3. Sensitivity analyses of relative risks of IPV after restricting to studies reporting adjusted estimates.

Supplementary Table 4. Sensitivity analyses of relative risks of IPV after restricting to studies adjusting for current age or studies only including participants of similar age.

Supplementary Table 5. Sensitivity analyses of IPV prevalence after applying transformations to prevalence estimates.

**Supplementary Methods. Search strategies.**

PubMed:

#1 "marital violence" or "marital abuse" or "marital aggression" or "spousal violence" or "spousal abuse" or "spousal aggression" or "partner violence" or "partner abuse" or "partner aggression" or "domestic violence" or "domestic abuse" or "domestic aggression" or "family violence" or "family abuse" or "family aggression"

#2 "child bride*" or "girl* bride*" or "teen* bride*" or "child marriage*" or "children marriage*" or "early marriage*" or "girl* marriage*" or "adolescen* marriage*" or "youth marriage*" or "teen* marriage*" or " married child" or "married children" or "married girl*" or "married adolescen*" or "married youth*" or "married teen*" or "child mother*" or "children mother*" or "early mother*" or "girl* mother*" or "adolescen* mother*" or "teen* mother*"

#3 #1 AND #2 Filters: Humans

EMBASE, APA PsycArticles, APA PsycInfo

1 ("marital violence" or "marital abuse" or "marital aggression" or "spousal violence" or "spousal abuse" or "spousal aggression" or "partner violence" or "partner abuse" or "partner aggression" or "domestic violence" or "domestic abuse" or "domestic aggression" or "family violence" or "family abuse" or "family aggression").tw.

2 ("child bride*" or "girl* bride*" or "teen* bride*" or "child marriage*" or "children marriage*" or "early marriage*" or "girl* marriage*" or "adolescen* marriage*" or "youth marriage*" or "teen* marriage*" or " married child" or "married children" or "married girl*" or "married adolescen*" or "married youth*" or "married teen*" or "child mother*" or "children mother*" or "early mother*" or "girl* mother*" or "adolescen* mother*" or "teen* mother*").tw.

3 1 and 2

4 limit 3 to human

Web of Science Core Collection

1. ALL=("marital violence" or "marital abuse" or "marital aggression" or "spousal violence" or "spousal abuse" or "spousal aggression" or "partner violence" or "partner abuse" or "partner aggression" or "domestic violence" or "domestic abuse" or "domestic aggression" or "family violence" or "family abuse" or "family aggression")

2. ALL=("child bride*" or "girl* bride*" or "teen* bride*" or "child marriage*" or "children marriage*" or "early marriage*" or "girl* marriage*" or "adolescen* marriage*" or "youth marriage*" or "teen* marriage*" or " married child" or "married children" or "married girl*" or "married adolescen*" or "married youth*" or "married teen*" or "child mother*" or "children mother*" or "early mother*" or "girl* mother*" or "adolescen* mother*" or "teen* mother*")

3. #1 AND #2

EBSCO:

CINAHL, Child Development & Adolescent Studies

S1 TX "marital violence" or "marital abuse" or "marital aggression" or "spousal violence" or "spousal abuse" or "spousal aggression" or "partner violence" or "partner abuse" or "partner aggression" or "domestic violence" or "domestic abuse" or "domestic aggression" or "family violence" or "family abuse" or "family aggression"

S2 TX "child bride*" or "girl* bride*" or "teen* bride*" or "child marriage*" or "children marriage*" or "early marriage*" or "girl* marriage*" or "adolescen* marriage*" or "youth marriage*" or "teen* marriage*" or " married child" or "married children" or "married girl*" or "married adolescen*" or "married youth*" or "married teen*" or "child mother*" or "children mother*" or "early mother*" or "girl* mother*" or "adolescen* mother*" or "teen* mother*"

S3 S1 AND S2 Limiters – Human

ProQuest Dissertations & Theses Global

(ti("marital violence" OR "marital abuse" OR "marital aggression" OR "spousal violence" OR "spousal abuse" OR "spousal aggression" OR "partner violence" OR "partner abuse" OR "partner aggression" OR "domestic violence" OR "domestic abuse" OR "domestic aggression" OR "family violence" OR "family abuse" OR "family aggression") OR ab("marital violence" OR "marital abuse" OR "marital aggression" OR "spousal violence" OR "spousal abuse" OR "spousal aggression" OR "partner violence" OR "partner abuse" OR "partner aggression" OR "domestic violence" OR "domestic abuse" OR "domestic aggression" OR "family violence" OR "family abuse" OR "family aggression")) AND (ti(("child bride" OR "child brides") OR "girl* bride*" OR "teen* bride*" OR ("child marriage" OR "child marriages") OR "children marriage*" OR ("early marriage" OR "early marriages") OR "girl* marriage*" OR "adolescen* marriage*" OR "youth marriage*" OR "teen* marriage*" OR " married child" OR "married children" OR ("married girl" OR "married girlfriend" OR "married girls") OR "married adolescen*" OR "married youth*" OR "married teen*" OR ("child mother") OR ("children mother") OR ("early mother") OR "girl* mother*" OR "adolescen* mother*" OR "teen* mother*") OR ab(("child bride" OR "child brides") OR "girl* bride*" OR "teen* bride*" OR ("child marriage" OR "child marriages") OR "children marriage*" OR ("early marriage" OR "early marriages") OR "girl* marriage*" OR "adolescen* marriage*" OR "youth marriage*" OR "teen* marriage*" OR " married child" OR "married children" OR ("married girl" OR "married girlfriend" OR "married girls") OR "married adolescen*" OR "married youth*" OR "married teen*" OR ("child mother") OR ("children mother") OR ("early mother") OR "girl* mother*" OR "adolescen* mother*" OR "teen* mother*"))

**Supplementary Table 1. Risk of bias assessment of included studies.**

| **Author (year)** | **1. Was the sample frame appropriate to address the target population?** | **2. Were study participants sampled in an appropriate way?** | **3. Was the sample size adequate?** | **4. Were the study subjects and the setting described in detail?** | **5. Was the data analysis conducted with sufficient coverage of the identified sample?** | **6. Were valid methods used for the identification of the condition?** | **7. Was the condition measured in a standard, reliable way for all participants?** | **8. Was there appropriate statistical analysis?** | **9. Was the response rate adequate, and if not, was the low response rate managed appropriately?** | **Overall quality** |
| --- | --- | --- | --- | --- | --- | --- | --- | --- | --- | --- |
| Yount et al. (2016) | yes | yes | yes | yes | na | yes | yes | yes | yes | high |
| Jabbi et al. (2020) | yes | yes | yes | yes | na | yes | yes | yes | yes | high |
| Gebrezgi et al. (2017) | no | no | no | yes | na | no | yes | yes | yes | low |
| Yüksel-Kaptanoğlu et al. (2012) | yes | yes | yes | yes | na | yes | yes | yes | yes | high |
| Ahinkorah et al. (2022) | yes | yes | yes | yes | na | yes | yes | yes | unclear | high |
| Kidman (2017) | yes | yes | yes | yes | na | yes | yes | yes | unclear | high |
| Kimuna et al. (2012) | yes | yes | yes | yes | na | yes | yes | yes | yes | high |
| Phuntsho et al. (2022) | yes | yes | yes | yes | na | yes | yes | yes | yes | high |
| Speizer & Pearson (2011) | yes | yes | yes | yes | na | yes | yes | yes | yes | high |
| Falb et al. (2015) | yes | no | no | yes | na | yes | yes | yes | unclear | low |
| Islam (2021) | no | no | no | no | na | yes | yes | yes | yes | low |
| Kidman & Heymann (2018) | yes | yes | yes | no | na | yes | yes | yes | unclear | high |
| Kiragu et al. (2022) | yes | yes | yes | yes | na | yes | yes | yes | unclear | high |
| Rahman et al. (2014) | yes | yes | yes | yes | na | yes | yes | yes | yes | high |
| El-Gazzar et al. (2020) | no | yes | no | yes | na | no | yes | yes | yes | moderate |
| Santhya et al. (2010) | no | yes | yes | yes | na | no | yes | yes | yes | moderate |
| Nasrullah et al. (2014) | yes | yes | no | yes | na | yes | yes | yes | yes | high |
| Qamar et al. (2022) | yes | yes | yes | yes | na | yes | yes | yes | unclear | high |
| Verma & Nair (2022) | yes | yes | yes | yes | na | yes | yes | yes | yes | high |
| Abera et al. (2020) | no | yes | yes | yes | na | unclear | yes | no | unclear | moderate |
| Olamijuwon et al. (2017) | yes | yes | yes | no | na | yes | yes | yes | unclear | high |
| Clark et al. (2017) | yes | yes | yes | yes | na | yes | yes | no | unclear | high |
| Durğut & Kısa (2018) | no | no | no | yes | na | no | yes | no | yes | low |
| Gubi et al. (2020) | yes | yes | yes | yes | na | yes | yes | yes | unclear | high |
| Mondal & Paul (2021a) | yes | yes | yes | yes | na | yes | yes | yes | yes | high |
| Mondal & Paul (2021b) | yes | yes | yes | yes | na | yes | yes | yes | yes | high |
| Oshiro et al. (2011) | no | yes | no | yes | na | yes | yes | yes | yes | moderate |
| Tenkorang (2019) | yes | yes | yes | yes | na | unclear | yes | yes | unclear | moderate |
| Valan & Srinivasan (2021) | no | no | no | yes | na | no | yes | no | yes | low |
| Erulkar (2013) | no | yes | yes | yes | na | no | yes | yes | unclear | moderate |
| Begum et al. (2015) | no | yes | yes | yes | na | yes | yes | yes | unclear | moderate |
| Huber-Krum et al. (2023) | yes | yes | yes | yes | na | no | yes | yes | yes | moderate |
| Mehari et al. (2023) | no | no | no | yes | na | unclear | yes | no | unclear | low |
| Silverman et al. (2023) | no | yes | yes | yes | na | yes | yes | no | yes | moderate |
| Verma & Choudhury (2023) | yes | yes | yes | no | na | yes | yes | yes | no | high |
| Soylu & Ayaz (2013) | no | no | no | yes | na | no | yes | no | yes | low |
| Patel et al. (2024) | no | yes | yes | yes | na | yes | yes | no | unclear | moderate |
| Seifu et al. (2024) | yes | yes | yes | no | na | yes | yes | no | unclear | high |

**Supplementary Fig. 1. Sensitivity analyses of lifetime IPV after restricting to nationally representative samples.**


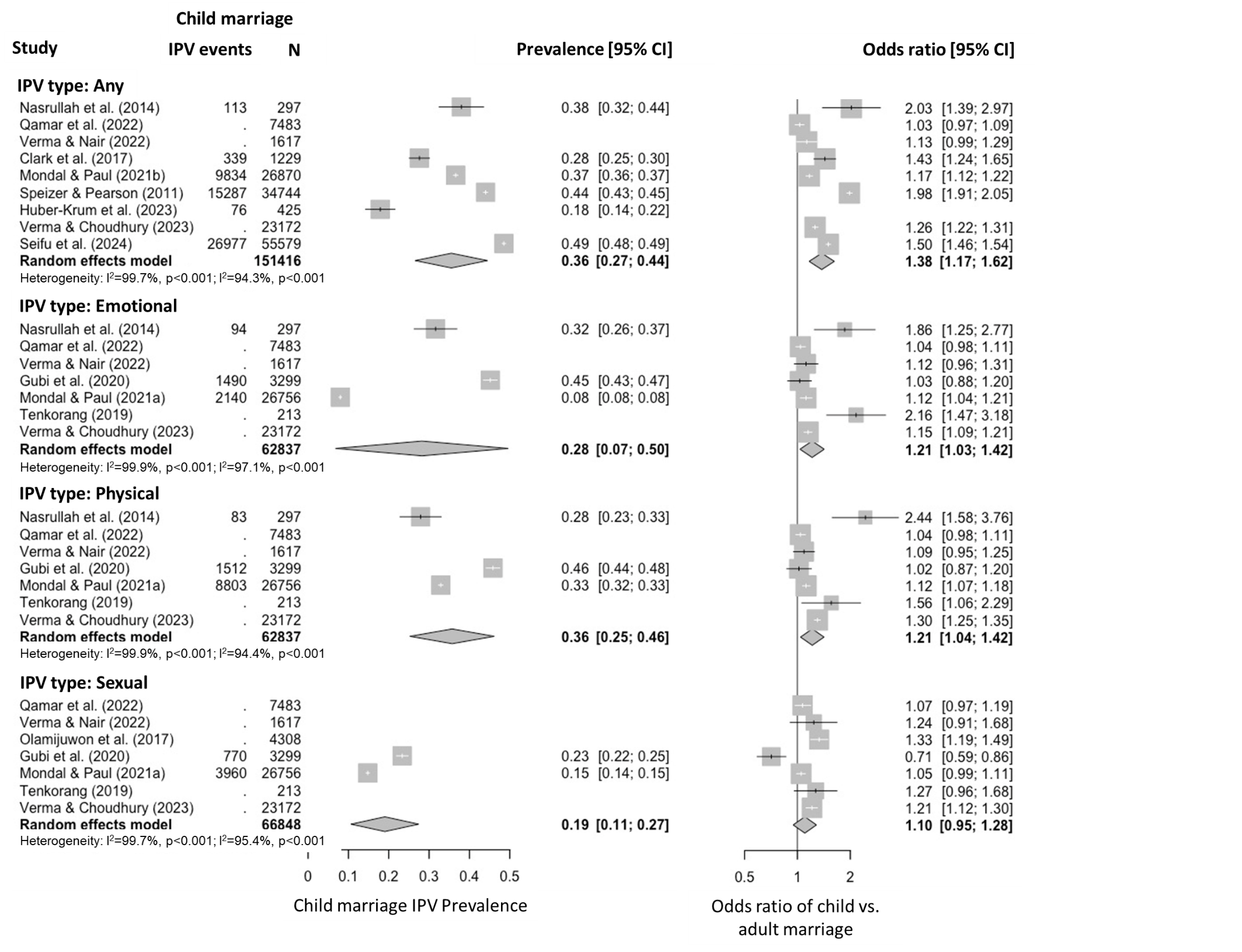


**Supplementary Fig. 2. Sensitivity analyses of 12-month IPV after restricting to nationally representative samples.**


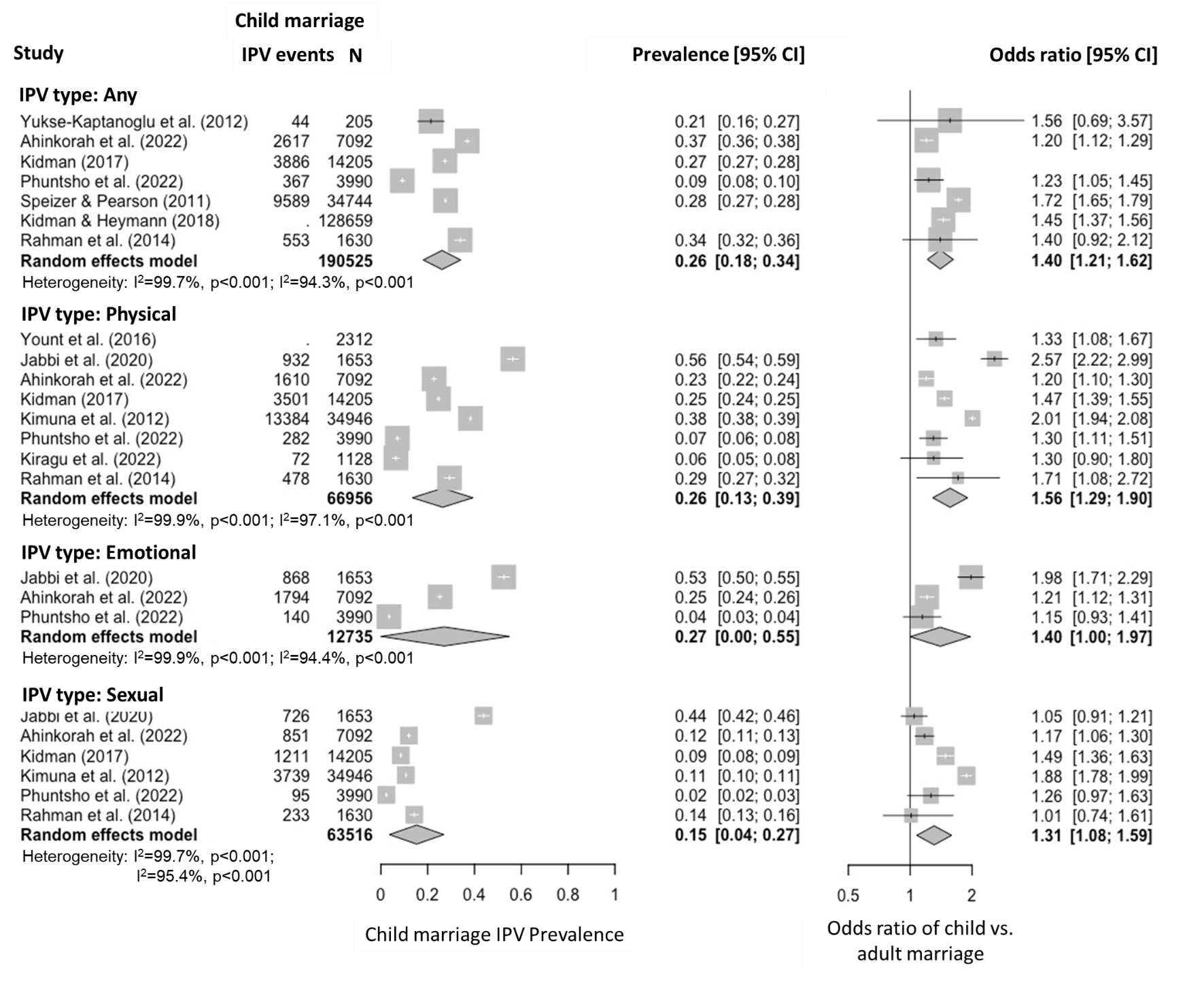


**Supplementary Fig. 3. DOI plots of the prevalence meta-analyses with at least 10 studies.**


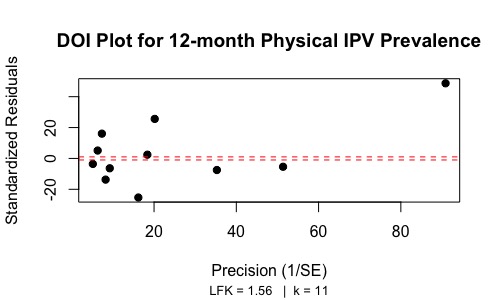


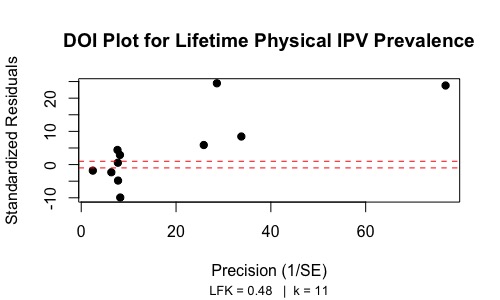


**Supplementary Fig. 4. Funnel plots of the relative-risk meta-analyses with at least 10 studies.**


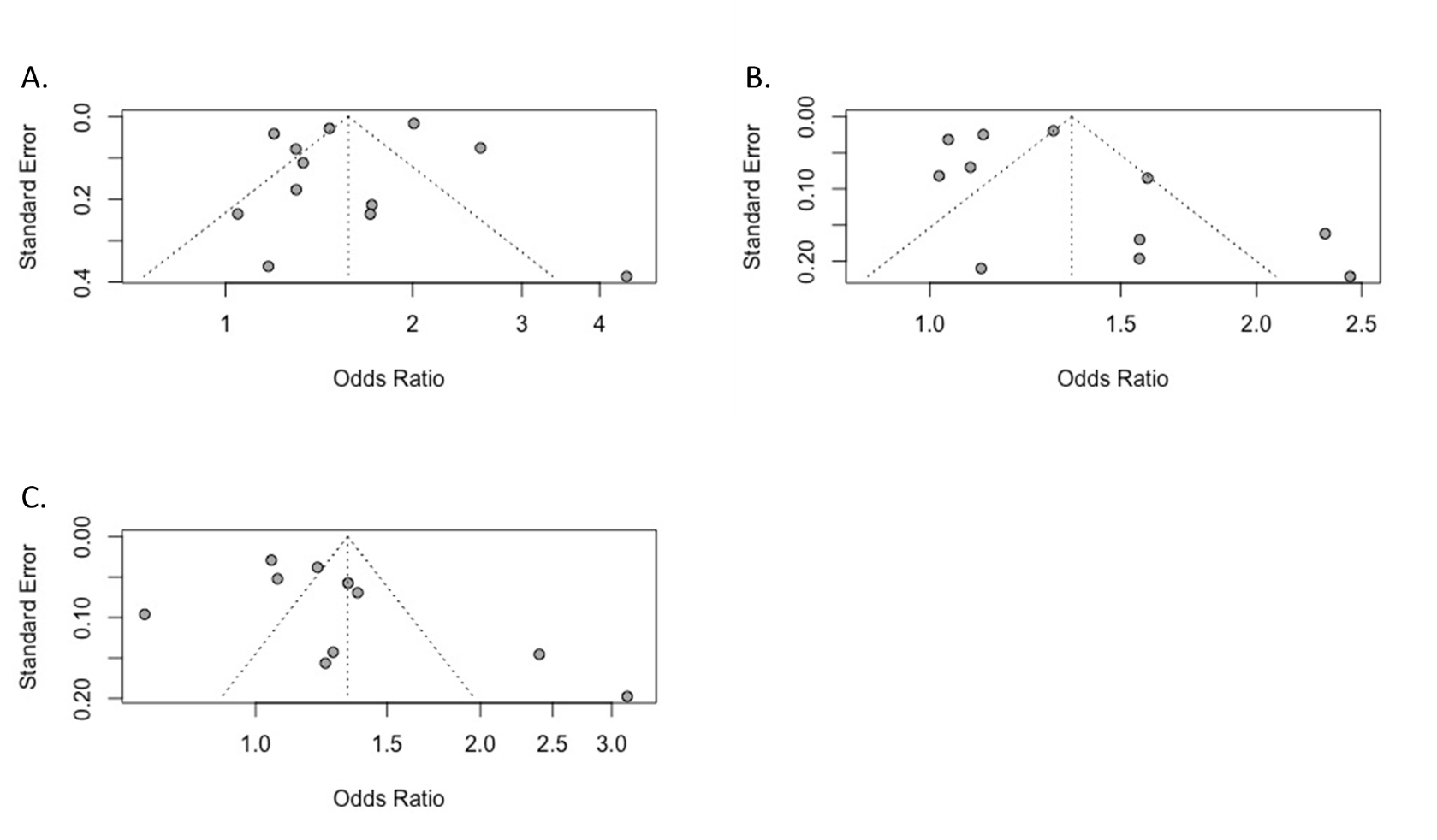


Note. Plot A: 12-month physical IPV odds ratio (k=12, Egger’s test *P*=0.350); Plot B: lifetime physical IPV odds ratio (k=11, Egger’s test *P*=0.372); Plot C: lifetime sexual IPV odds ratio (k=10, Egger’s test *P*=0.155).

**Supplementary Table 2. Sensitivity analyses after restricting to studies rated as high quality.**

| **Prevalence** | **IPV type** | **k** | **Estimates** | **95% CI** |
| --- | --- | --- | --- | --- |
| 12 months | Any | 6 | 0.26 | [0.18; 0.34] |
|  | Emotional | 3 | 0.27 | [0.00; 0.55] |
|  | Physical | 7 | 0.26 | [0.13; 0.39] |
|  | Sexual | 6 | 0.15 | [0.04; 0.27] |
| lifetime | Any | 5 | 0.39 | [0.31; 0.46] |
|  | Emotional | 3 | 0.28 | [0.07; 0.50] |
|  | Physical | 3 | 0.36 | [0.25; 0.46] |
|  | Sexual | 2 | 0.19 | [0.11; 0.27] |
| **Odds ratio** |  |  |  |  |
| 12 months | Any | 6 | 1.40 | [1.21; 1.62] |
|  | Emotional | 3 | 1.40 | [1.00; 1.90] |
|  | Physical | 8 | 1.57 | [1.29; 1.92] |
|  | Sexual | 6 | 1.31 | [1.08; 1.60] |
| lifetime | Any | 8 | 1.38 | [1.17; 1.62] |
|  | Emotional | 6 | 1.11 | [1.05; 1.17] |
|  | Physical | 6 | 1.18 | [1.02; 1.37] |
|  | Sexual | 6 | 1.08 | [0.91; 1.28] |

**Supplementary Table 3. Sensitivity analyses of relative risks of IPV after restricting to studies reporting adjusted estimates.**

|  | **IPV type** | **k** | **Odds ratio for child vs. adult marriages** | **95% CI** |
| --- | --- | --- | --- | --- |
| 12 months | Any | 5 | 1.31 | [1.17; 1.47] |
|  | Emotional | - | - | - |
|  | Physical | 5 | 1.65 | [1.24; 2.20] |
|  | Sexual | 3 | 1.14 | [0.87; 1.49] |
| lifetime | Any | 5 | 1.20 | [1.06; 1.36] |
|  | Emotional | 7 | 1.21 | [1.03; 1.42] |
|  | Physical | 8 | 1.27 | [1.08; 1.49] |
|  | Sexual | 8 | 1.13 | [0.98; 1.31] |

Note: “-” refers to no analysis being conducted due to low number of studies in certain categories.

**Supplementary Table 4. Sensitivity analyses of relative risks of IPV after restricting to studies adjusting for current age or studies only including participants of similar age.**

|  | **IPV type** | **k** | **Odds ratio for child vs. adult marriages** | **95% CI** |
| --- | --- | --- | --- | --- |
| 12 months | Any | 4 | 1.33 | [1.15; 1.55] |
|  | Emotional | - | - | - |
|  | Physical | 6 | 1.37 | [1.22; 1.54] |
|  | Sexual | 4 | 1.27 | [1.07; 1.51] |
| lifetime | Any | 3 | 1.29 | [0.90; 1.85] |
|  | Emotional | 5 | 1.30 | [0.98; 1.73] |
|  | Physical | 7 | 1.29 | [1.05; 1.59] |
|  | Sexual | 6 | 1.25 | [0.86; 1.81] |

Note: “-” refers to no analysis being conducted due to low number of studies in certain categories.

**Supplementary Table 5. Sensitivity analyses of IPV prevalence after applying transformations to prevalence estimates.**

| **12 months** | **IPV type** | **k** | **prevalence** | **95% CI** |
| --- | --- | --- | --- | --- |
| Freeman–Tukey double-arcsine transformation | Any | 8 | 0.23 | [0.16; 0.31] |
|  | Emotional | 4 | 0.27 | [0.08; 0.53] |
|  | Physical | 11 | 0.29 | [0.17; 0.43] |
|  | Sexual | 9 | 0.18 | [0.08; 0.31] |
| Logit transformation | Any | 8 | 0.22 | [0.15; 0.31] |
|  | Emotional | 4 | 0.23 | [0.07; 0.57] |
|  | Physical | 11 | 0.27 | [0.15; 0.42] |
|  | Sexual | 9 | 0.16 | [0.08; 0.29] |
| **lifetime** | | | | |
| Freeman–Tukey double-arcsine transformation | Any | 8 | 0.35 | [0.27; 0.43] |
|  | Emotional | 7 | 0.30 | [0.19; 0.43] |
|  | Physical | 11 | 0.26 | [0.20; 0.34] |
|  | Sexual | 6 | 0.26 | [0.19; 0.34] |
| Logit transformation | Any | 8 | 0.35 | [0.28; 0.43] |
|  | Emotional | 7 | 0.31 | [0.21; 0.43] |
|  | Physical | 11 | 0.27 | [0.21; 0.34] |
|  | Sexual | 6 | 0.26 | [0.20; 0.34] |
